# Elucidating the Role of the Mixing Entropy in Equilibrated Nanoconfined Reactions

**DOI:** 10.3390/e27060564

**Published:** 2025-05-27

**Authors:** Leonid Rubinovich, Micha Polak

**Affiliations:** Department of Chemistry, Ben-Gurion University of the Negev, Beer-Sheva 8410500, Israel; rubin@bgu.ac.il

**Keywords:** mixing entropy, reaction nanosystem, thermal fluctuations, reaction extent, equilibrium statistical mechanics, canonical partition function

## Abstract

By introducing the concept of nanoreaction-based fluctuating mixing entropy, the challenge posed by the smallness of a closed molecular system is addressed through equilibrium statistical–mechanical averaging over fluctuating reaction extent. Based on the canonical partition function, the interplay between the mixing entropy and fluctuations in the reaction extent in nanoscale environments is unraveled while maintaining consistency with macroscopic behavior. The nanosystem size dependence of the mixing entropy, the reaction extent, and a concept termed the “reaction extent entropy” are modeled for the combination reactions A+B↔2C and the specific case of $H_{2}+I_{2}↔2HI$. A distinct inverse correlation is found between the first two properties, revealing consistency with the nanoconfinement entropic effect on chemical equilibrium (NCECE). To obtain the time dependence of the instantaneous mixing entropy following equilibration, the Stochastic Simulation (Gillespie) Algorithm is employed. In particular, the smallest nanosystems exhibit a step-like behavior that deviates significantly from the smooth mean values and is associated with the discrete probability distribution of the reaction extent. As illustrated further for molecular adsorption and spin polarization, the current approach can be extended beyond nanoreactions to other confined systems with a limited number of species.

## 1. Introduction

Nanoconfined chemical reactions, namely those occurring inside completely closed nanoscopic space [1] (only heat can be exchanged with the external environment) exhibit distinct properties compared to macroscopic reaction systems. (The same term, “nanoconfinement”, is often used in catalysis [2], namely for semi-open chemical systems.). A variety of molecular “nanoreactors” fabricated for such confined reactions were reported, including self-assembling molecular flasks [3], cages for nucleotide dimerization [4], capsules for cycloaddition reactions [5], ssDNA hybridization inside nanofabricated chambers [6], and biological nanosystems, etc. Theoretical treatments of chemical equilibrium in small closed systems focused on reversible association reactions (and their kinetics) in droplets [7], general association reactions [8], dimerization [9], multimerization and aggregation reactions [10], and adsorption in small closed systems [11]. As tentatively suggested in our previous studies [12,13,14], the mixing entropy, which is a somewhat controversial concept [15], can play a distinct role in equilibrated nanoconfined reactions. The role of the mixing entropy in the thermodynamic limit (TL) of macroscopic systems is better established [16]. In particular, it is responsible for the occurrence of backward reactions; namely, in many cases, the reaction extent does not reach completion, despite the product’s nonmixing free energy being lower than the reactant value [17]. While this reaction incompleteness is attributed to the reactant–product mixing entropy, the latter can be strongly diminished or even absent in the case of nanoconfined reactions, shifting exothermic ones forward [12] (backward shift in the case of endothermicity). This predicted phenomenon was originally termed by us the “nanoconfinement entropic effect on chemical equilibrium”, NCECE [12,13]. Despite the suggested intuitive link between the effect and mixing entropy variations, no formulation of the entropic effect has been derived. Moreover, the few later studies that addressed the issue of equilibrated nanoconfined reactions [8,9,11] did not consider the possible role of the mixing entropy. Hence, the primary motivation behind the present study was triggered by the lack of an appropriate methodology for quantitatively assessing the role of the mixing entropy in the reaction shift. It can be noted that in the case of isomerization reactions, Hill predicted no difference between the reaction extent of small closed systems and the macroscopic behavior [18], while in bi-molecular reactions, the NCECE effect is expected to emerge [12,13].

This entropic effect is quite general in the sense that it solely originates from the smallness of the molecular system, while the confinement effects of energetic origins, such as system interactions with the confined space boundaries, as well as geometrical constraints, are beyond the scope of this research. Thus, the main focus of this study is on the interplays between the mixing entropy and fluctuations in the reaction extent and its mean value. For this goal, a formulation of the mixing entropy that is specifically pertinent to nanoconfined reaction systems is introduced below.

Starting with the TL, the well-known expression for the mixing entropy, i.e., the difference between the mixed and unmixed systems, reads [16] as follows:

(1) $$S_{mix}^{TL}\equiv S-S^{\left(0\right)}=-kN\sum_{i}\chi_{i}ln\chi_{i}$$

where $\chi_{i}$ is the mole fraction of constituent i in the mixture, N is the total number of molecules, and k is the Bolzmann constant. However, in nanosystems, this expression poses some difficulties, because both the mixed state and the corresponding initial unmixed state display notable fluctuations in the reaction extent and the associated mole fractions. Consequently, as noted above, one of the primary goals of this study is to resolve the issue via statistical mechanics and establish a procedure for a quantitative evaluation of the mixing entropy’s role in nanoconfined reactions (Section 2.1). In addition, fluctuation-induced variabilities in the mixing entropy and the reaction extent as a function of the equilibrium constant are fully addressed (Section 2.2). The role of the mixing entropy for the specific case of $H_{2}+I_{2}↔2HI$ is given in Section 2.3, and for nanoconfined adsorption and spin ½ polarization in Section 2.4. Finally, the issue of post-equilibration instantaneous (versus mean) values is addressed in Section 3. Inspection of this stochastic equilibrium further illustrates the role of the mixing entropy in nanoconfined reaction systems (it can be distinguished from “stochastic thermodynamics” that deal with non-equilibrated mesoscopic systems [19,20,21]).

## 2. Statistical Mechanics Modeling and Computations

The classical thermodynamics of macroscopically large systems is not fully applicable to small groups of molecules or atoms, so a modified formalism was proposed by Hill [18] and used in later studies, e.g., in [22]. Equilibrium statistical mechanics constitutes an alternative approach that is also valid for small systems [18]. In particular, the canonical partition function is suitable for handling fluctuations under nanoconfinement [12].

### 2.1. Nanoconfined Reaction Mixing Entropy

The elemental chemical reaction is generally expressed as follows:

$$\sum r_{i}R_{i}=0,$$

where $R_{i}$ stands for the reagents and $r_{i}$ denotes the associated stoichiometric coefficients. The coefficients $r_{i}$ are negative for reactants and positive for products.

The canonical partition function of the nanoreaction mixture M closed within volume V is given by the following:

(2) $$Z=\sum_{x}Z^{\left(x\right)},$$

where $Z^{\left(x\right)}$ is the partition function of the subsystem $M^{\left(x\right)}$, corresponding to the reaction extent x. The reaction extent, undergoing a discrete set of values, is defined by the relation $x=\frac{X}{X_{max}}$, where $X=0,1,2,\ldots ,X_{max}$ marks the number of forward reaction steps. $Z^{\left(x\right)}$ for an ideal gas reaction mixture includes the reagent contributions corresponding to a certain x value:

(3) $$Z^{\left(x\right)}=e^{-\frac{xX_{max}\Delta E}{kT}}\prod_{i}\frac{{\left(V\lambda_{i}^{-3}z_{i}^{int}\right)}^{\chi_{x,i}N_{x}}}{\left(\chi_{x,i}N_{x}\right)!}.$$

Here, ΔE is the reaction energy, $\lambda_{i}=\frac{h}{\sqrt{2\pi m_{i}kT}}$ is the thermal de Broglie wavelength related to the translational contribution to the partition function, and $z_{i}^{int}$ is the internal molecular partition function [16]. $N_{x}$ and $\chi_{x,i}\left(=\chi_{0,i}+r_{i}x\frac{X_{max}}{N_{x}}\right)$ are the total number of molecules and the molar fraction of the i-reagent, respectively. Thus, the probability of the reaction extent reads as follows:

(4) $$u\left(x\right)=\frac{Z^{\left(x\right)}}{Z}.$$

**Figure 1:**
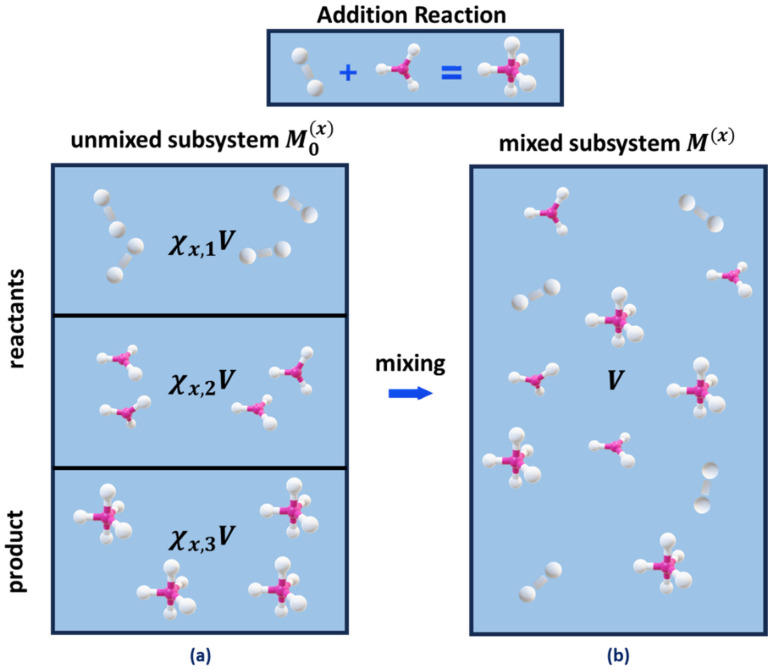
Schematic representation of reagent mixing for an addition reaction: (**a**) unmixed subsystem with separate compartments for each gas reagent, with $\chi_{x,i}$ denoting the mole fraction of the i-th reagent at reaction extent x and $\chi_{x,i}V$—the volume of the i-th compartment, and (**b**) the mixed subsystem with total volume V containing all reagents.

In addition to the mixed subsystem $M^{\left(x\right)}$, we consider a hypothetical unmixed subsystem $M_{0}^{\left(x\right)}$, which contains the same number of reactant and product molecules, but each gas occupies its initial compartment of volume $\chi_{x,i}V$ (Figure 1). The partition function of this unmixed subsystem $M_{0}^{\left(x\right)}$ is as follows:

$$Z_{0}^{\left(x\right)}=e^{-\frac{xX_{max}\Delta E}{kT}}\prod_{i}\frac{{\left(\left(\chi_{x,i}V\right)\lambda_{i}^{-3}z_{i}^{int}\right)}^{\chi_{x,i}N_{x}}}{\left(\chi_{x,i}N_{x}\right)!},$$

which simplifies to the following:

(5) $$Z_{0}^{\left(x\right)}=Z^{\left(x\right)}\exp\left(N_{x}\sum_{i}\chi_{x,i}ln\chi_{x,i}\right).$$

The difference between the entropies of $M^{\left(x\right)}$ and $M_{0}^{\left(x\right)}$ defines the mixing entropy at reaction extent x, and is given by (Appendix A.1):

(6) $$S_{mix}^{\left(x\right)}\equiv S^{\left(x\right)}-S_{0}^{\left(x\right)}=-kN_{x}\sum_{i}\chi_{x,i}ln\chi_{x,i}.$$

It can be noted that $S_{mix}^{\left(x\right)}$ reflects the expansion of the reagents from compartment volumes $\chi_{x,i}V$ to the whole volume V [15] (Figure 1), and that Equation (6) has a form similar to Equation (1) for macroscopic systems.

Since both $\chi_{x,i}$ and $S_{mix}^{\left(x\right)}$ fluctuate with the reaction extent x, we have chosen to define the mixing entropy by means of its mean value:

(7) $$S_{mix}\equiv \sum_{x}u\left(x\right)S_{mix}^{\left(x\right)}.$$

This accounts for the inherent variability in the fluctuating mixing entropy, providing a better representation than evaluating the mixing entropy at the mean reaction extent:

(8) $$\xi \equiv \sum_{x}u\left(x\right)x.$$

According to Jensen’s inequality [23], due to the concavity of the function $S_{mix}^{\left(x\right)}$ with respect to x, the mean value $S_{mix}$ is smaller than $S_{mix}^{\left(\xi \right)}$. In macroscopic systems, the extent probability distribution $u\left(x\right)$ sharply peaks, so that the mean mixing entropy $S_{mix}$ coincides with $S_{mix}^{\left(TL\right)}$ given by Equation (1).

The total entropy of a closed system is given by the following [24]:

(9) $$S=k\left(lnZ+T\frac{\partial }{\partial T}lnZ\right),$$

Using Equations (2) and (4) gives the following:

(10) $$S=k\left(lnZ-\sum_{x}u\left(x\right)lnZ^{\left(x\right)}\right)+\sum_{x}u\left(x\right)S^{\left(x\right)},$$

where $S^{\left(x\right)}=lnZ^{\left(x\right)}+T\frac{\partial }{\partial T}lnZ^{\left(x\right)}$ consistently with Equation (9). Using $\sum_{x}u\left(x\right)=1$ and Equation (6), we obtain the following:

$$S=-k\sum_{x}u\left(x\right)\ln\left[u\left(x\right)\right]+\sum_{x}u\left(x\right)S_{mix}^{\left(x\right)}+\sum_{x}u\left(x\right)S_{0}^{\left(x\right)}.$$

Thus, we find that the total entropy of the nanoconfined reaction mixture consists of three contributions:

(11) $$S=S_{ree}+S_{mix}+S_{0}.$$

$S_{0}\equiv \sum_{x}u\left(x\right)S_{0}^{\left(x\right)}$ is the mean value of the entropy of the unmixed system, and the “reaction extent entropy” introduced here reads as follows:

(12) $$S_{ree}\equiv -k\sum_{x}u\left(x\right)\ln\left[u\left(x\right)\right].$$

This contribution reflects the degree of randomness in the reaction extent, revealing in what manner it is spread across its possible values (“x related microstates”).

According to Equation (11), the difference in entropy between the mixed and unmixed nanosystems reads as follows:

(13) $$\Delta S\equiv S-S_{0}=S_{ree}+S_{mix}.$$

The contribution from the fluctuating reaction extent $S_{ree}$ appears to be relatively marginal (computed below).

For computational convenience, the partition function can be expressed in terms of the equilibrium constant K (Appendix A.2):

(14) $$Z=\sum_{x}\left({\left(\frac{K}{\kappa }\right)}^{xX_{max}}\prod_{i}\frac{1}{\left(\chi_{x,i}N_{x}\right)!}\right),$$

where the coefficient $\kappa \equiv {\left(N_{Av}V_{L}\right)}^{-\sum_{i}r_{i}}$ converts the number of reagent molecules to moles per unit volume, $V_{L}$, given in liters ($N_{Av}$ is the Avogadro’s number). Consistently with Equations (2) and (4), the extent probability distribution is expressed as follows:

(15) $$u\left(x\right)=\frac{1}{Z}{\left(\frac{K}{\kappa }\right)}^{xX_{max}}\prod_{i}\frac{1}{\left(\chi_{x,i}N_{x}\right)!}.$$

This form of the partition function (14) is quite general in the sense that it does not explicitly depend on the molecular partition function specific to each reagent. It is used in the next section to compute the extent of a particular nanoconfined reaction, the mixing and reaction extent entropies as a function of the equilibrium constant K.

### 2.2. The General Combination Reaction A+B↔2C

**Figure 2:**
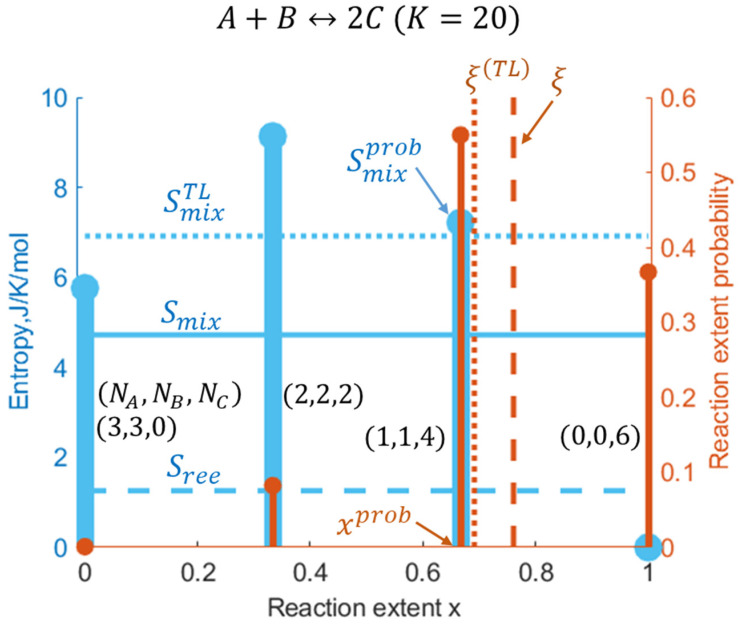
Stem plots of discrete reaction extent probabilities (orange) and the corresponding mixing entropy (blue) of the nanoconfined reaction. The number of the three reagent molecules is given in parentheses (the initial composition is stoichiometric). Dotted, solid, and dashed horizontal lines denote the TL, mean, and reaction extent entropy levels, respectively.

This nanoconfined combination reaction has been chosen for concrete computations, starting from the partition function Equation (14) and the extent probability distribution Equation (15) depicted in Figure 2. They include the mean reaction extent ξ, the mean mixing entropy $S_{mix}$, and the reaction extent entropy $S_{ree}$, calculated using Equations (7), (8), and (12), respectively. Consistently with the nanoconfinement entropic effects on chemical equilibrium (NCECE) [12,13], the mean reaction extent shifts towards the product side compared to the TL value, as the number of mixed “levels” between reactants and products, and thus the mean mixing entropy, decreases. Despite this equilibrium shift in ξ, there remains some residual probability for reactants due to the thermal fluctuations inherent in nanosystems and the related $S_{ree}$ (which is quite small, Figure 2). Additionally, the skewness of the probability distribution results in significant deviations in the predicted mean values from the most probable values of both the reaction extent and the mixing entropy ($x^{prob}$ and $S_{mix}^{prob}$). While the mean values represent a comprehensive measure considering all possible x-values, the most probable values provide the most frequent, typical values. Consequently, the mean characteristics are more relevant for long time measurements, whereas the most probable ones are pertinent for short time scales.

**Figure 3:**
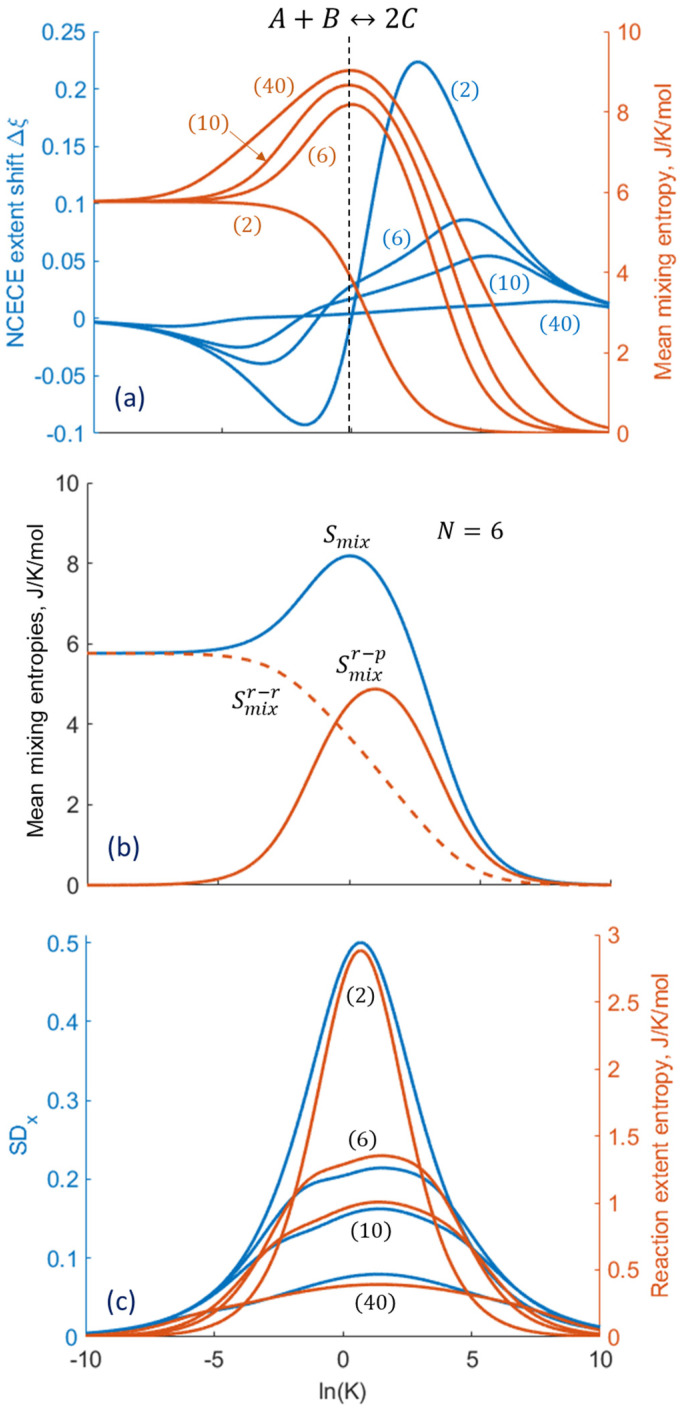
Equilibrium properties of the stoichiometric A+B↔2C nanoconfined reaction as a function of the equilibrium constant K. (**a**) Mean mixing entropy and NCECE extent shift in the mean reaction extent (Δξ) for different initial numbers of reactant molecules $\left(N\right)$. (**b**) $S_{mix}$ (blue line), its decomposition to $S_{mix}^{r-p}$ (orange line), and $S_{mix}^{r-r}$ (orange dashed line) for six-molecule nanoconfined reaction. (**c**) Reaction extent entropy and standard deviation.

Using the same computational procedure, the mean values and standard deviations are obtained as function of K for different nanosystem sizes (Figure 3). The NCECE shift in the mean reaction extent, $\Delta \xi \equiv \xi -\xi^{TL}$, is positive mostly in the exothermic region (K>1), while an inverse NCECE [25] is predicted for K<1. For K≈1, i.e., when the molar fractions of reagents A, B, and C are similar, Δξ approaches zero and the mixing entropy reaches maximal values (Figure 3a). The smallest two-molecular system shows no maximum due to the absence of reactant–product mixing. For the same reason, the $S_{mix}$ plot minima converge at the extreme K values.

It should be noted that $S_{mix}$, given by Equations (6) and (7), consists of two components, namely, the reactant–product mixing entropy, $S_{mix}^{r-p}$ and the reactant–reactant mixing contribution, $S_{mix}^{r-r}$ (Appendix A.3). The former component, $S_{mix}^{r-p}$, which is directly relevant to the NCECE effect, exhibits a nearly symmetric peak, while $S_{mix}^{r-r}$ forms a monotonically decreasing background. This background introduces asymmetry into the overall $S_{mix}$ profile around K=1 (Figure 3a,b). The rest of the following computations are focused on $S_{mix}$ alone. The inclusion of $S_{mix}^{r-r}$ as part of this overall mixing entropy marginally affects the magnitude of the predicted results, without changing the general trend.

The reaction extent standard deviation ($SD_{x}$) and entropy ($S_{ree}$, which is generally smaller than the mixing entropy, see, e.g., Figure 2) display similar shapes and size-dependent variations (Figure 3c). This occurs because both originate from the thermal fluctuations in the reaction extent, and contrary to the $S_{mix}$ behavior, their maxima become lower as the number of molecules (nanosystem size) increases. As can be seen, these properties become negligible at the extreme K values, as do the associated fluctuations.

**Figure 4:**
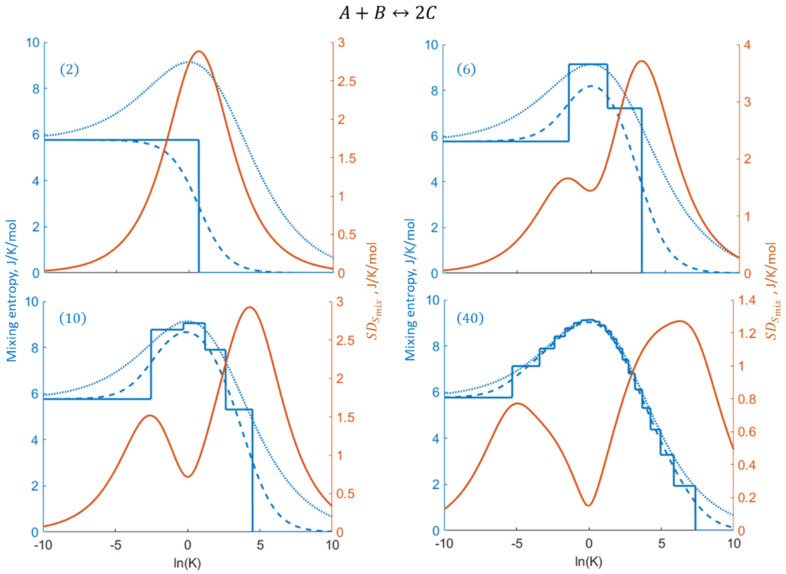
For increasing nanosystem size $\left(N\right)$, the four panels display $S_{mix}^{prob}$ (blue solid lines), $S_{mix}$ (blue dashed lines), $S_{mix}^{TL}$ (blue dotted lines), and $SD_{S_{mix}}$ (orange solid lines) as functions of lnK (the colors of the different lines match those of the axes).

Comparisons of the different mixing entropy characteristics and the fluctuations expressed by means of the standard deviations vs. *K* are shown for the same nanoconfined reactions in Figure 4. In larger systems, just like the reaction extent, the mixing entropy $S_{mix}$ gradually approaches the thermodynamic limit value, $S_{mix}^{TL}$. The step-like behavior of the most probable mixing entropy $S_{mix}^{prob}$ deviates significantly from the smoother $S_{mix}$ curves in smaller nanosystems due to larger reaction extent steps, but gradually converges with them as the system size increases. Except for the smallest system $\left(2\right)$, $SD_{S_{mix}}$ increases with the decreased system size due to increasing fluctuations. In larger nanosystems, the emergence of an $SD_{S_{mix}}$ minimum signifies a decrease in the variability of $S_{mix}$, associated with the local flatness of $S_{mix}$ at its maximum for K≈1.

For the smallest two-molecule nanosystem, analytical relationships hold between $S_{mix}$ and $S_{ree}$ versus ξ (which is related to K). The relationships are given by $S_{mix}=2kln2\left(1-\xi \right)$ and $S_{ree}=-k\left[\left(1-\xi \right)ln\left(1-\xi \right)+\xi ln\xi \right]$. For relatively large nanosystems, the discrete probability distribution of the reaction extent x can be approximated by a continuous Gaussian probability density function (Appendix A.4):

$$f\left(x\right)\approx \sqrt{\frac{N}{2\pi \xi \left(1-\xi \right)}}\exp\left(-\frac{N{\left(x-\xi \right)}^{2}}{2\xi \left(1-\xi \right)}\right).$$

with the standard deviation as follows:

$$SD_{x}=\sqrt{\frac{\xi \left(1-\xi \right)}{N}}.$$

This approximation, which becomes more accurate as the number of molecules N increases while remaining in the nanoscale regime, is used here to transform Formulas (7) and (12) from a discrete to continuous representation. This allows us to explore the asymptotic behavior of $S_{mix}$ and $S_{ree}$:

$$S_{mix}\approx -kN\left(\left(1-\xi \right)ln\left(\frac{1-\xi }{2}\right)+\xi ln\left(\xi \right)\right)-\frac{k}{2},$$

$$S_{ree}\approx \frac{k}{2}\ln\left(\frac{e}{2}\pi \xi \left(1-\xi \right)N\right).$$

where e is Euler’s number. $S_{ree}\ll S_{mix}$ for large N, because the former depends on $\ln\left(N\right)$ compared to a linear dependence of $S_{mix}$.

For a specific reaction of interest (e.g., in Section 2.3), K in Formula (14) can be expressed in terms of the individual molecular partition functions of the reagents (Appendix A.2):

(16) $$K=\kappa V^{\sum_{i}r_{i}}e^{-\frac{\Delta E}{kT}}\prod_{i}{\left(\lambda_{i}^{-3}z_{i}^{int}\right)}^{r_{i}}.$$

According to expressions (14) and (16), the partition function-based calculations generally depend on V. Examples of this are the addition reaction A+B↔C and the analogous non-dissociative adsorption (Section 2.4). However, in the present case of A+B↔2C, $\sum_{i}r_{i}=0$; namely, the total number of molecules remains unchanged during the reaction, so the calculated results should be independent of the system’s volume. (Nonetheless, because a small number of molecules typically occupies a nanoscopic volume, the term “nanoconfinement” is used also in this case.).

### 2.3. Specific Example: $H_{2}+I_{2}↔2HI$

The model system selected for analysis focuses on the temperature and size dependencies of the mixing and extent entropies in the nanoconfined gaseous reaction $H_{2}+I_{2}↔2HI$, which is treated as an elementary direct process (other possible mechanisms [26] have been excluded for the sake of simplicity). This reaction is treated within the framework of the rigid rotor harmonic oscillator (RRHO) approximation [24]. The internal molecular partition function of the i-th reagent under the RRHO approximation is given by the following equation:

$$z_{i}^{int}=\frac{T}{\sigma_{i}\Theta_{i}^{rot}}\frac{\exp\left(\frac{D_{i}}{kT}\right)}{1-\exp\left(-\frac{\Theta_{i}^{vib}}{T}\right)},$$

where $D_{i}$ is the dissociation energy, $\sigma_{i}$ is the symmetry number, $\Theta_{i}^{rot}$ is the characteristic rotational temperature, and $\Theta_{i}^{vib}$ is the characteristic vibrational temperature. The input data for these calculations are given in Table 1.

**Figure 5:**
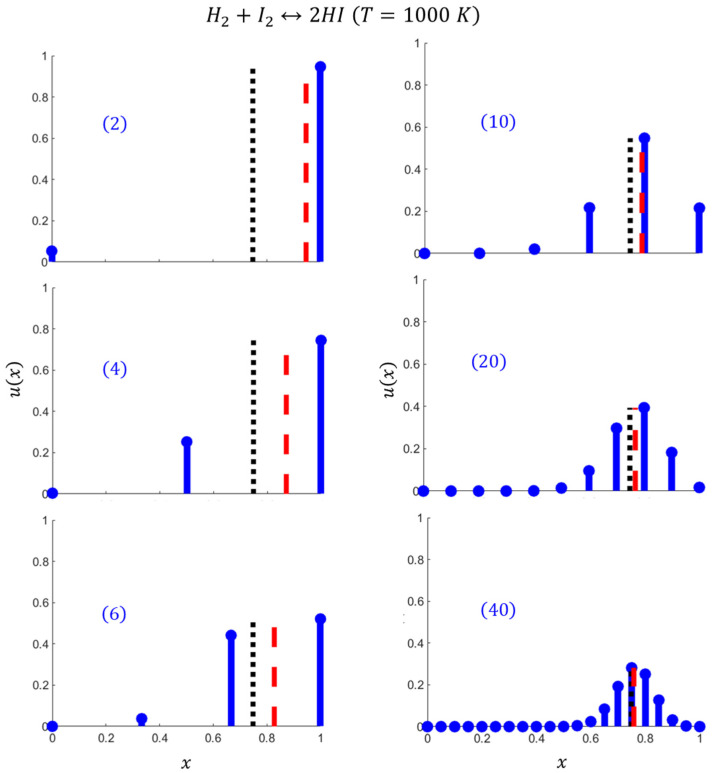
The reaction extent probability distributions (blue stem plots) computed for six stoichiometric nanosystem sizes $\left(N\right)$. Red dashed lines: the mean reaction extents $\left(\xi \right)$. Black dotted line: the TL reaction extent.

**Figure 6:**
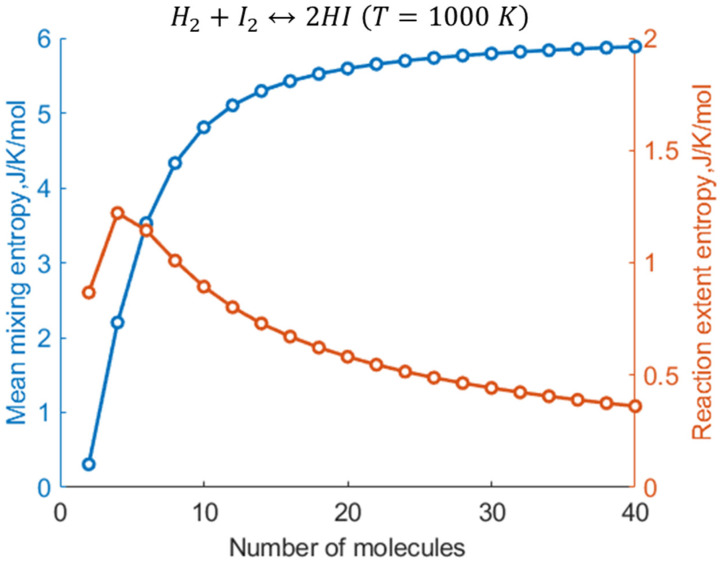
The dependence of $S_{mix}$ and $S_{ree}$ on the nanosystem size.

**Figure 7:**
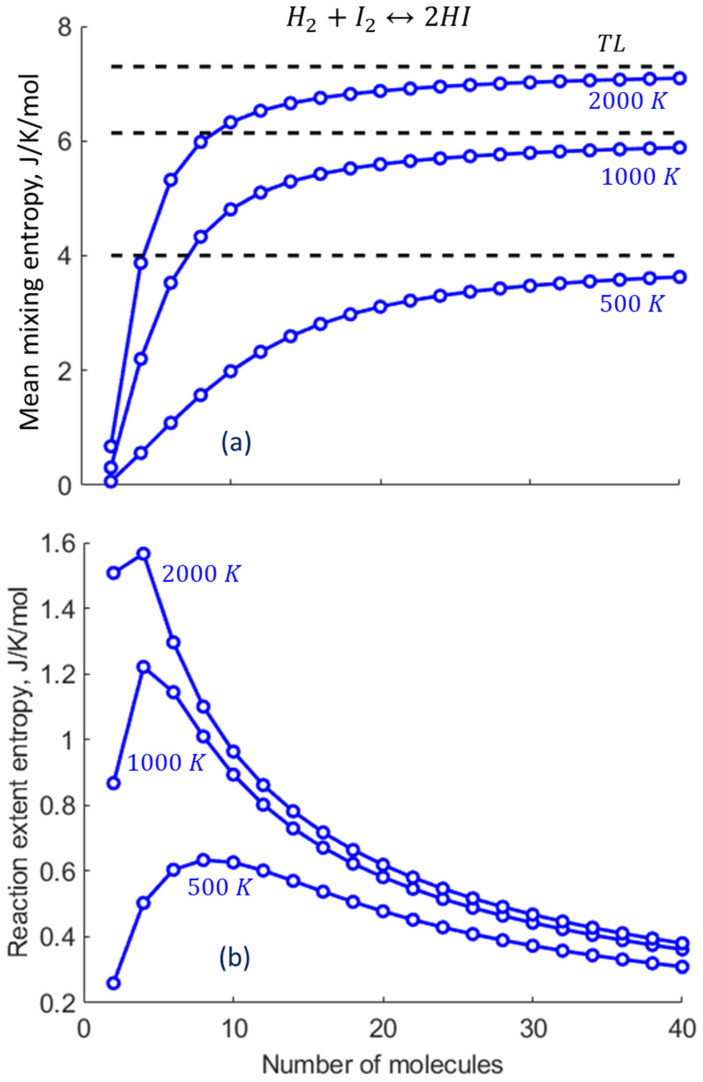
The dependence of (**a**) $S_{mix}$ and (**b**) $S_{ree}$ on the nanosystem size computed for three temperatures.

The calculations based on Equations (14)–(16) show that reducing the number of molecules in the system lowers the number of mixed “levels”, i.e., the levels shared between the reactants and products, thereby gradually shifting the mean reaction extent away from the thermodynamic limit (the NCECE effect), as shown in Figure 5 (see also Figure 2 in Section 2.2). The effect is most remarkable in the smallest nanosystem (2), since the absence of mixed levels makes level x=1 highly probable, driving the reaction nearly to completion consistently with the NCECE. Moreover, level x=1 contributes almost exclusively to the summations in Equations (7) and (12), resulting in low values of both $S_{mix}$ and the slightly higher $S_{ree}$ (Figure 6). They exhibit a different dependence on the system size; namely, $S_{mix}$ gradually increases with N, approaching the TL, while $S_{ree}$ decreases after an initial rise. For N>4, $S_{mix}$ significantly exceeds $S_{ree}$, the latter being constrained by both the narrowing of the $u\left(x\right)$ distribution and its reduced magnitude with increased size (Figure 5). With increasing temperature, both $S_{mix}$ and $S_{ree}$ become larger, reflecting the general entropic trend of enhanced mixing and greater fluctuations in the reaction extent (Figure 7).

**Figure 8:**
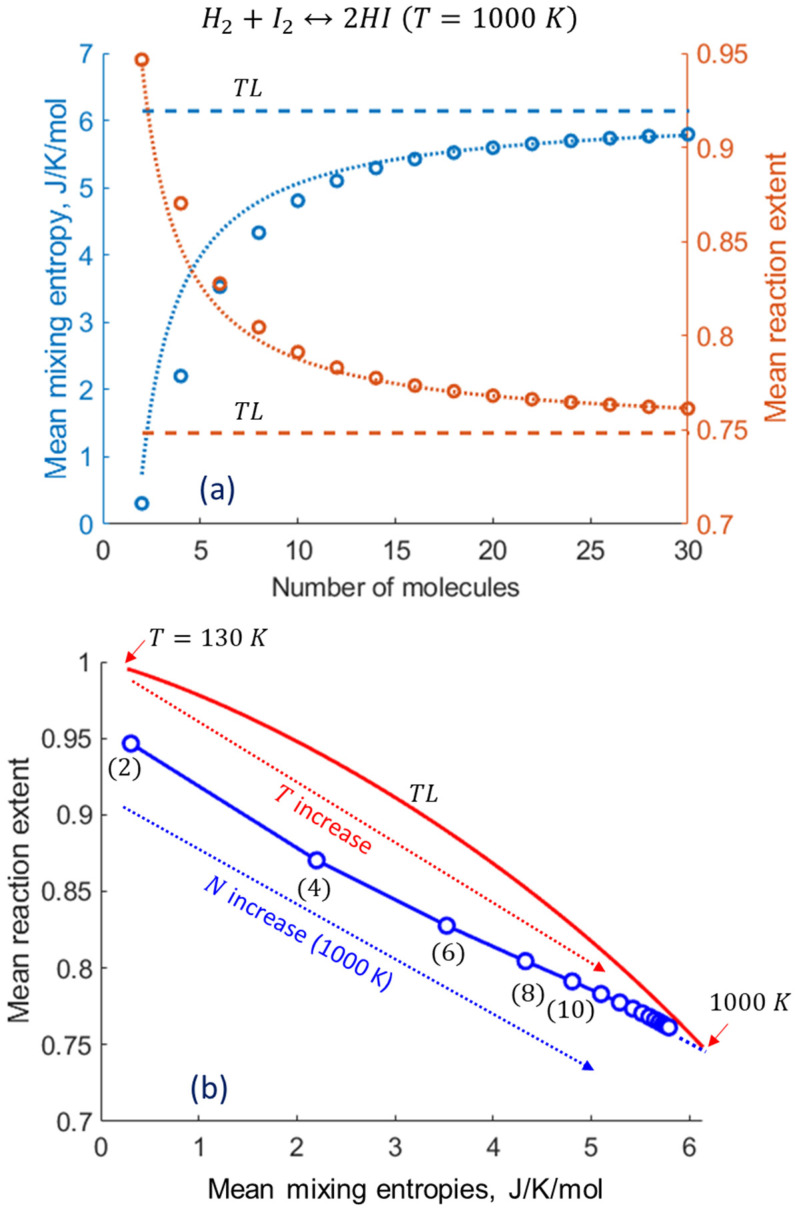
Inverse correlation between the mean mixing entropy $S_{mix}$ and the mean reaction extent ξ, reflecting the NCECE origin. (**a**) The computed dependence of $S_{mix}$ and ξ on N is represented by circles. The opposite shifts relative to the TL values ($\Delta S_{mix}$ and Δξ) are almost proportional to 1/N (dotted lines). (**b**) The dependence of ξ on $S_{mix}$ originated from nanosystem size variations and from temperature variations (in the TL).

As we intuitively assumed earlier [12], the mixing entropy plays a distinct role in the predicted shift in the nanoconfined reaction extent. Indeed, the observed reduction in the mixing entropy, as shown in Figure 8a, is in inverse correlation with the enhancement of the reaction extent, approaching the thermodynamic limit as the system size grows. Thus, this finding quantitatively establishes the origin of the “nanoconfinement entropic effect on chemical equilibrium” (NCECE). Furthermore, the shifts in both the mean mixing entropy $\left(\Delta S_{mix}\right)$ and the mean reaction extent $\left(\Delta \xi \right)$ from their TL values scale inversely with the system size, particularly for large N (Figure 8a). This reflects their apparent proportionality to the decreasing step width Δx of the reaction extent, namely, its narrowing as the system approaches macroscopic quasi-continuity. In macrosystems, an inverse correlation between the mixing entropy decrease and exothermic reaction extent enhancement can be driven by temperature reduction, whereas nanosystems exhibit this inverse correlation even at constant temperatures (Figure 8b). The increase in the TL reaction extent induced by the lowered temperature somewhat exceeds that induced by the system size decrease, and the overall curve shape differs, namely, concave vs. convex, respectively.

### 2.4. The Role of the Mixing Entropy in Other Nanosystems

The above introduced approach to the mixing entropy effects can be extended to other confined systems with a limited number of species. As an illustration, two phenomena are analyzed: molecular adsorption and spin polarization.

*Adsorption under nanoconfinement:* The non-dissociative equilibrated adsorption of a few molecules (N) onto the same number of surface sites is explored. Previously [27], the effect of nanoconfinement on typically exothermic adsorption (NCEA) was predicted using a combined framework of the ideal gas and lattice gas models, assuming equivalent adsorption sites and no interactions between adsorbates. Specifically, it was shown that, compared to the TL, the adsorbate coverage significantly increased. A clear formal analogy can be drawn between equilibrated molecular adsorption on a surface and addition reactions; namely, desorbed molecules and vacant adsorption sites (“vacancies”) can be considered reactants, and the adsorbed molecules are the products, while the surface coverage x corresponds to the reaction extent. The mixing entropy arises solely from the different possible arrangements of the adsorbed molecules and vacancies, while the desorbed molecules remain unmixed in the gas phase.

The mixing entropy at a given coverage x, as derived in Appendix A.5, reads as follows:

$$S_{mix}^{\left(x\right)}=kln\frac{N!}{\left(Nx\right)!\left(N-Nx\right)!}.$$

For macroscopic systems N→∞, so the probability distribution of the coverage sharply peaks around the mean coverage θ. In this thermodynamic limit, the mixing entropy can be expressed by the known formula:

$$S_{mix}^{TL}=-kN\left(\theta ln\theta +\left(1-\theta \right)\ln\left(1-\theta \right)\right)$$

**Figure 9:**
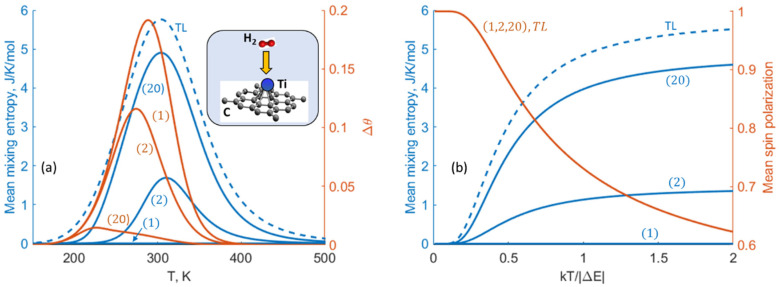
(**a**) Temperature dependence of $S_{mix}$ and $H_{2}$ mean coverage shift ($\Delta \theta \equiv \theta -\theta^{\left(TL\right)}$) computed for three system sizes (N) and the thermodynamic limit (TL) in the case of a single molecule per Ti surface site in graphene-like nanostructures. (Preadsorption constant volume $=10^{3}\;nm^{3}$ per molecule). Inset: schematics of the smallest nanosystem (N=1). A uniform input adsorption-energy of 0.407 eV per $H_{2}$ [28] is assumed for all adsorption bonds. (**b**) Temperature dependence of the mean mixing entropy and mean spin polarization computed for three spin system sizes discussed below.

Similarly to the combination reaction analyzed above (Figure 3), the mean mixing entropy for $H_{2}$ on Ti-doped graphene-like nanostructures [27] decreases significantly with decreasing system size, while the mean coverage increases; namely, the two are inversely correlated (Figure 9a).

*Spin 1/2 system*: In the present context, such a system is analogous to the A↔B isomerization reaction associated with its similar two-state nature. In the case of overall N spins on a lattice having energy difference ΔE<0 between spin down and up states with respect to an external magnetic field, the mixing entropy at spin polarization x is given by the following (Appendix A.6):

$$S_{mix}^{\left(x\right)}\equiv kln\frac{N!}{\left(Nx\right)!\left(N-Nx\right)!},$$

which formally coincides with the adsorption case.

For macroscopic systems, in which the probability distribution sharply peaks around the mean spin polarization P, the mixing entropy is given by the known formula:

$$S_{mix}^{TL}=-kN\left(PlnP+\left(1-P\right)\ln\left(1-P\right)\right).$$

The mean mixing entropies computed according to Equation (7) show a significant decrease as the system size is reduced (Figure 9b), like the general trend predicted above for the combination reaction and adsorption. However, in the spin system, an NCECE-like effect on the mean spin polarization is absent, consistent with its absence in isomerization reactions [12,18]. This is due to individual spin flips (or isomerization) occurring independently of neighbors, which consequently leads to insensitivity to the system size, namely, $P=\frac{1}{1+e^{\frac{\Delta E}{kT}}}$ (Appendix A.6). This demonstrates that, depending on the specific system, the nanoconfinement effect on the mixing entropy is not always accompanied by an NCECE-like effect.

## 3. Instantaneous Mixing Entropy and Extent in Equilibrated A+B↔2C

**Figure 10:**
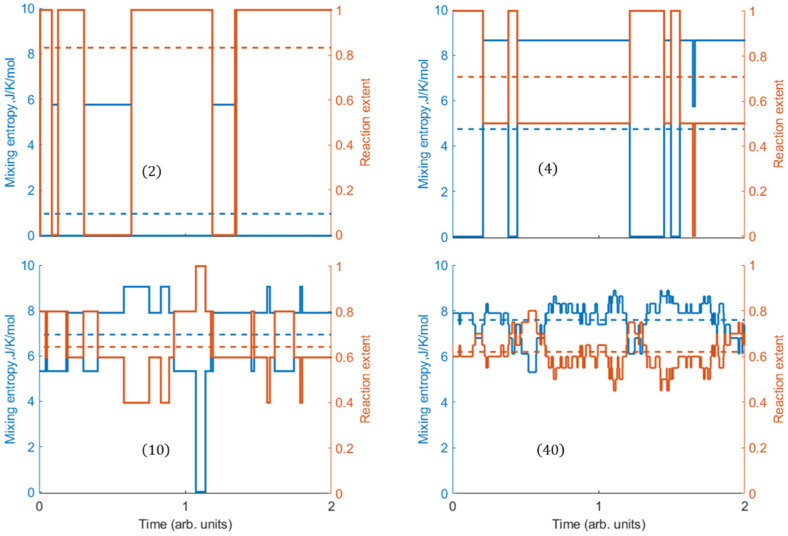
Computed stepwise time dependencies of the instantaneous reaction extent (orange) and mixing entropy (blue) for one of the realizations of the A+B↔2C reaction generated with the SSA, compared to the equilibrium mean values (dashed lines) based on the canonical partition function. $\left(N\right)$ marks the nanoreaction system size. Elementary rate constants for the forward and backward reactions are $k_{f}=10$ and $k_{b}=1$, respectively (K=10).

In view of the computed significant fluctuations in the mixing entropy and extent occurring at the equilibrium state, we explored the instantaneous values relative to their respective means over extended timescales. These values do not change during the short time intervals between consecutive reaction steps, which correspond to a certain reaction extent x and the respective mixing entropy $S_{mix}^{\left(x\right)}$ (Figure 10). These typical single realizations of the stoichiometric combination reaction for different nanosystem sizes were simulated using the Monte Carlo method, known as Gillespie’s Stochastic Simulation Algorithm (SSA) [29]. The ability of the SSA to take proper account of the discrete, stochastic nature of chemical reactions makes it well suited to systems containing small numbers of molecules [30]. While the SSA is primarily employed to simulate stochastic kinetics, in the present simulations time counting begins after the stochastic equilibrium in the system has been well established. (When using the SSA, time can be measured in arbitrary units in models where the exact time scale is not essential for understanding the system behavior.). For all sizes, there is a clear inverse correlation between the instantaneous mixing entropy and reaction extent values.

**Figure 11:**
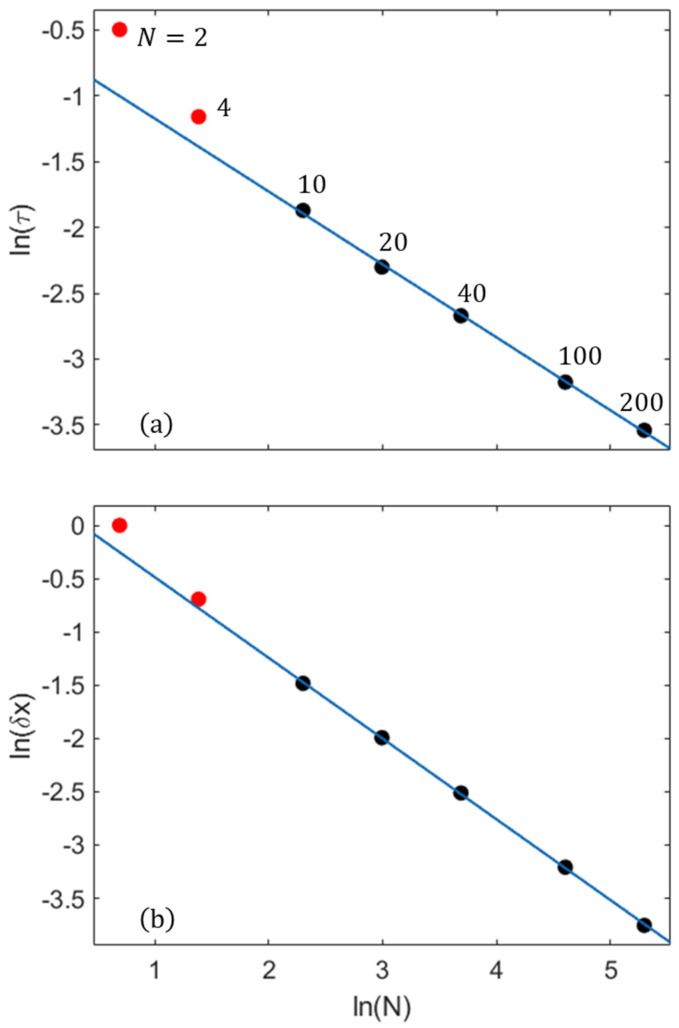
Scaling plots for the A+B=2C post-equilibration behavior: (**a**) the mean recurrence period (τ) and (**b**) the mean maximal deviation amplitude (δx) of the instantaneous reaction extent. N is the total number of molecules. (More than $10^{4}$ steps were included in the τ and δx computations for each size.).

The nanosystems comprising only a few molecules exhibit significant deviations in the instantaneous values from the mean values, whereas for N = 40, the deviations are minor. As can be seen (Figure 10), the instantaneous characteristics restore to their prior state within a limited number of steps during a recurrence period (*τ*). In smaller nanosystems, reductions in the numbers of molecule pairs lowers the overall frequency of reaction steps, resulting in an increased period *τ*. (In an SSA simulation, smaller numbers of reacting molecule pairs result in lower propensities.). The amplitude δx, defined by the mean maximal deviation of the instantaneous *x* from the value nearest to the mean extent ξ during this period, also increases with decreased nanosystem size. This is due to the exponential increase in the probability of large fluctuations (e.g., [31]). As seen in Figure 11, both τ and δx obey exponential scaling laws. In particular, based on the data acquired from ${>10}^{4}$ steps of the SSA for each system size, the fitted dependences for N≳10 molecules are expressed as $\tau \propto e^{-0.55N}$ and $\delta x\propto e^{-0.76N}.$ In very small systems, the decrease in the number of neighboring collision partners leads to an additional increase in τ and, consequently, in *δx*, both deviating from the scaling line (Figure 11, N = 2, 4). The instantaneous extent variations can probably be detected using high-spatial- and temporal-resolution techniques [32,33,34].

In addition to the reaction extent *x*, each realization is characterized by the associated mixing entropy $S_{mix}^{\left(x\right)}$, which also exhibits stepwise and recurrent behavior (Figure 10). Clearly, the two properties are inversely correlated. While $S_{mix}^{prob}$ shown in Figure 4 is also stepwise, it is time independent, as it refers to the peak of the equilibrium probability distribution $u\left(x\right)$, whereas $S_{mix}^{\left(x\right)}$ follows the random variations in the instantaneous reaction extent.

Considering the possible observation of reaction extent fluctuations in nanosystems, the results of short time measurements can significantly differ, even under the seemingly static conditions of chemical equilibrium. Furthermore, each measurement is expected to reveal a specific reaction extent, similarly to measurements in quantum mechanics that determine a particular system state. This uncertainty in the reaction extent (of the order δx), as well as in the associated energy, somewhat resembles the quantum mechanical uncertainty principle, although the two are obviously not related at the basic physical level. However, longer measurement durations should average out the fluctuations, resulting in minimal deviations from the mean value. These perspectives emphasize the influence of the observation period on the measured properties of stochastic reaction nanomixtures. It can be noted that quantum techniques were incorporated in studies on equilibrium in complex reaction networks [35].

## 4. Summary

This study introduces a formulation of the mixing entropy that is specifically pertinent to nanoreaction systems. Via statistical–mechanical analysis using the canonical partition function, we investigate the distinct roles of the mixing entropy and of the newly introduced “reaction-extent entropy”, and find how the mean and most probable mixing entropy, along with the standard deviations, vary with the system size and the equilibrium constant, particularly in the context of the general combination reaction A+B↔2C. Additionally, examination of the reaction $H_{2}+I_{2}↔2HI$ demonstrates how the system size and temperature govern the entropic contributions. Perhaps the most important result of this study is the inverse correlation found between the mean mixing entropy and the mean reaction extent, furnishing quantitative insights into the “nanoconfinement entropic effects on chemical equilibrium” (NCECE) reported by us before. In particular, the reduced mixing entropy in small reaction mixtures hinders the backward reaction and so leads to a forward extent shift towards the products.

In a separate section, the Gillespie’s Stochastic Simulation Algorithm (SSA) is used to calculate the instantaneous mixing entropy, which corresponds to the instantaneous reaction extent, as a function of time (following equilibration) for different nanosystem sizes. Their step-like time dependence deviates significantly from the constant mean values, especially for the smaller nanosystems. These findings regarding the fluctuating extent and mixing entropy along with their instantaneous values form a basis for future research on nanoreaction systems through both computational modeling and advanced techniques, such as high-resolution electron microscopy and ultrashort pulsed electron beam diffraction.

As demonstrated by nanoconfined adsorption and spin polarization, the present approach to the treatment of entropic contributions is applicable to a broad range of other confined systems characterized by a limited number of species. Finally, issues to be explored elsewhere include the transferability of the different entropy formulas introduced in this study for equilibrated nanoconfined reactions to the case of non-equilibrium conditions, and the role of the mixing entropy in nanoconfined reaction kinetics.

## Figures and Tables

**Table 1:** RRHO input data [24].

Reagent Molecule	D, eV *	σ	$\Theta^{rot}$, K	$\Theta^{vib}$, K
$H_{2}$	4.48	2	85.3	6215
$I_{2}$	1.55	2	0.0537	308
HI	3.06	1	9.06	3266

* Reaction energy $\Delta E=-\left(2D_{HI}-D_{H_{2}}-D_{I_{2}}\right)=-96\;meV$.

## Data Availability

The original contributions presented in this study are included in the article. Further inquiries can be directed to the corresponding author(s).

## Appendix A

### Appendix A.1. Mixing Entropy for a Given Reaction Extent

The entropy of subsystem $M^{\left(x\right)}$ with a fixed number of reagent molecules at extent x is as follows [24]:

(A1) $$S^{\left(x\right)}=k\left(lnZ^{\left(x\right)}+T\frac{\partial }{\partial T}lnZ^{\left(x\right)}\right).$$

According to (5), the following can be calculated:

(A2) $$Z^{\left(x\right)}=Z_{0}^{\left(x\right)}\exp\left(-N_{x}\sum_{i}\chi_{x,i}ln\chi_{x,i}\right).$$

Placing $Z^{\left(x\right)}$ into (A1) and considering that for a given x, ${\left(\frac{\partial \chi_{x,i}}{\partial T}\right)}_{x}=0$, the following holds:

$$S^{\left(x\right)}=-kN_{x}\sum_{i}\chi_{x,i}ln\chi_{x,i}+S_{0}^{\left(x\right)},$$

where the entropy of the unmixed subsystem $M_{0}^{\left(x\right)}$ is as follows:

(A3) $$S_{0}^{\left(x\right)}=k\left(lnZ_{0}^{\left(x\right)}+T\frac{\partial }{\partial T}lnZ_{0}^{\left(x\right)}\right).$$

The differences between the entropies of the mixed and unmixed systems at reaction step x define the mixing entropy:

(A4) $$S_{mix}^{\left(x\right)}\equiv S^{\left(x\right)}-S_{0}^{\left(x\right)}=-kN_{x}\sum_{i}\chi_{x,i}ln\chi_{x,i}.$$

### Appendix A.2. The Relationship Between the Partition Function and the Equilibrium Constant

The derivation presented here is similar to the approach detailed in the Supporting Information of our previous study [14].

The canonical partition function of the nanoconfined reaction mixture of volume V is given as follows:

(A5) $$Z=\sum_{x}Z^{\left(x\right)}=\sum_{x}\left(e^{-\frac{xX_{max}\Delta E}{kT}}\prod_{i}\frac{{\left(V\lambda_{i}^{-3}z_{i}^{int}\right)}^{N_{x,i}}}{N_{x,i}!}\right),$$

where $N_{x,i}$ is the number of i-reagent molecules ($N_{x,i}=\chi_{x,i}N_{x}$) related to the initial number of i-reagent molecules $N_{0,i}$ and reaction extent x:

(A6) $$N_{x,i}=N_{0,i}+r_{i}xX_{max},$$

By substituting Equation (A6) into Equation (A5) and factoring out the common reaction extent independent term from the summation, the following is obtained:

(A7) $$Z=\prod_{i}{\left(V\lambda_{i}^{-3}z_{i}^{int}\right)}^{N_{i}^{\left(0\right)}}\sum_{x}\left(\left(\prod_{i}\frac{1}{N_{x,i}!}\right){\left(e^{-\frac{\Delta E}{kT}}V^{\sum_{i}r_{i}}\prod_{i}{\left(\lambda_{i}^{-3}z_{i}^{int}\right)}^{r_{i}}\right)}^{xX_{max}}\right).$$

Considering first the TL case, the maximal term in the partition function is highly dominant, and it corresponds to the following condition:

$$\frac{\partial }{\partial x}\left[\sum_{i}N_{i}^{\left(0\right)}ln\left(V\lambda_{i}^{-3}z_{i}^{int}\right)-\sum_{i}\ln\left(N_{x,i}!\right)+xX_{max}ln\left(e^{-\frac{\Delta E}{kT}}V^{\sum_{i}r_{i}}\prod_{i}{\left(\lambda_{i}^{-3}z_{i}^{int}\right)}^{r_{i}}\right)\right]=0.$$

Taking into account that $\frac{\partial \ln\left(N_{x,i}!\right)}{\partial N_{x,i}}=\ln\left(N_{x,i}\right)$ for large $N_{x,i}$ and $\frac{\partial N_{x,i}}{\partial x}=r_{i}X_{max}$, the following equation is obtained:

$$\sum_{i}r_{i}X_{max}\ln\left(N_{x,i}\right)=X_{max}ln\left(e^{-\frac{\Delta E}{kT}}V^{\sum_{i}r_{i}}\prod_{i}{\left(\lambda_{i}^{-3}z_{i}^{int}\right)}^{r_{i}}\right).$$

This simplifies further to the following:

(A8) $$\prod_{i}N_{x,i}^{r_{i}}=e^{-\frac{\Delta E}{kT}}V^{\sum_{i}r_{i}}\prod_{i}{\left(\lambda_{i}^{-3}z_{i}^{int}\right)}^{r_{i}}.$$

The value of the thermodynamic equilibrium constant, K, expressed in terms of molarity, is as follows:

(A9) $$K=\prod_{i}{\left(\frac{N_{x,i}}{V_{L}N_{Av}}\right)}^{r_{i}}=\kappa \prod_{i}N_{x,i}^{r_{i}}=\kappa e^{-\frac{\Delta E}{kT}}V^{\sum_{i}r_{i}}\prod_{i}{\left(\lambda_{i}^{-3}z_{i}^{int}\right)}^{r_{i}},$$

where $V_{L}=1000V$ is given in liters, $N_{Av}$ is Avogadro’s number, and the coefficient $\kappa \equiv {\left(V_{L}N_{Av}\right)}^{-\sum_{i}r_{i}}$ converts the number $N_{x,i}$ of $R_{i}$-molecules to moles per unit volume. From Equations (A8) and (A9), the following can be obtained:

(A10) $$\frac{K}{\kappa }=e^{-\frac{\Delta E}{kT}}V^{\sum_{i}r_{i}}\prod_{i}{\left(\lambda_{i}^{-3}z_{i}^{int}\right)}^{r_{i}}.$$

This is similar to the equilibrium constant expression from Ref. [16]. Using (A7) and (A10), the partition function can be rewritten as follows:

$$Z=\prod_{i}{\left(V\lambda_{i}^{-3}z_{i}^{int}\right)}^{N_{i}^{\left(0\right)}}\sum_{x}\left({\left(\frac{K}{\kappa }\right)}^{xX_{max}}\prod_{i}\frac{1}{N_{x,i}!}\right).$$

When calculating the nanosystem properties that are determined by the reaction extent, such as the probability of a specific reaction extent, the mixing entropy, and the entropy of the reaction extent, the reaction extent independent prefactor can be neglected. This allows for the following:

(A11) $$Z=\sum_{x}\left({\left(\frac{K}{\kappa }\right)}^{xX_{max}}\prod_{i}\frac{1}{\left(\chi_{x,i}N_{x}\right)!}\right).$$

### Appendix A.3. Decomposition of the Total Mixing Entropy into Reactant–Product and Reactant–Reactant Mixing Contributions for the A+B=2C General Reaction

According to Equations (6) and (7), the total mixing entropy is given as follows:

$$S_{mix}=-\sum_{x}u\left(x\right)kN\left(\chi_{A}\left(x\right)ln\chi_{A}\left(x\right)+\chi_{B}\left(x\right)ln\chi_{B}\left(x\right)+\chi_{C}\left(x\right)ln\chi_{C}\left(x\right)\right).$$

This expression can be decomposed into two components:

$$S_{mix}=S_{mix}^{r-p}+S_{mix}^{r-r}.$$

The mixing entropy reactant–product contribution is as follows:

$$S_{mix}^{r-p}=-\sum_{x}u\left(x\right)kN\left(\left(\chi_{A}\left(x\right)+\chi_{B}\left(x\right)\right)ln\left(\chi_{A}\left(x\right)+\chi_{B}\left(x\right)\right)+\chi_{C}\left(x\right)ln\chi_{C}\left(x\right)\right),$$

And the reactant–reactant contribution reads as follows:

$$S_{mix}^{r-r}=-\sum_{x}u\left(x\right)kN\left(\chi_{A}\left(x\right)+\chi_{B}\left(x\right)\right)\left(\frac{\chi_{A}\left(x\right)}{\chi_{A}\left(x\right)+\chi_{B}\left(x\right)}ln\frac{\chi_{A}\left(x\right)}{\chi_{A}\left(x\right)+\chi_{B}\left(x\right)}+\frac{\chi_{B}\left(x\right)}{\chi_{A}\left(x\right)+\chi_{B}\left(x\right)}ln\frac{\chi_{B}\left(x\right)}{\chi_{A}\left(x\right)+\chi_{B}\left(x\right)}\right).$$

Being directly related to the reaction extent, $S_{mix}^{r-p}$ is more relevant to the NCECE effect.

### Appendix A.4. Approximation Based on the Gaussian Probability Density

For relatively large nanosystems, the probability $u\left(x\right)$ is dominated by values of x very close to its maximum at x=ξ and exceedingly small steps of $\Delta x=\frac{2}{N}$ (in the present case of the stoichiometric reaction A+B↔2C). Near ξ, to a good approximation, $u\left(x\right)$ can be considered a continuous function. By expanding $ln\left[u\left(x\right)\right]$ to a second order around x=ξ, we obtain the following:

(A12) $$ln\left[u\left(x\right)\right]\approx ln\left[u\left(\xi \right)\right]+\frac{1}{2}{\left(\frac{d^{2}ln\left[u\left(x\right)\right]}{dx^{2}}\right)}_{x=\xi }{\left(x-\xi \right)}^{2}.$$

Considering Equations (3) and (4), and using Stirling’s approximation, the second derivative is given as follows:

(A13) $${\left(\frac{d^{2}ln\left[u\left(x\right)\right]}{dx^{2}}\right)}_{x=\xi }\approx -\frac{N}{\xi \left(1-\xi \right)}.$$

In the continuous representation, a probability density function $f\left(x\right)\approx \frac{u\left(x\right)}{\Delta x}$ can be used. Substituting in Equation (A12) and considering that the constant Δx does not affect the $ln\left[u\left(\xi \right)\right]$ derivative, the following approximate equation is obtained:

$$\ln\left[f\left(x\right)\right]\approx \ln\left[f\left(\xi \right)\right]-\frac{N}{2\xi \left(1-\xi \right)}{\left(x-\xi \right)}^{2},$$

which leads to the Gaussian function:

(A14) $$f\left(x\right)\approx \sqrt{\frac{N}{2\pi \xi \left(1-\xi \right)}}\exp\left(-\frac{N{\left(x-\xi \right)}^{2}}{2\xi \left(1-\xi \right)}\right),$$

with the following standard deviation:

(A15) $$SD_{x}=\sqrt{\frac{\xi \left(1-\xi \right)}{N}}.$$

The Gaussian approximation is used here to transform Formulas (7) and (12) for $S_{mix}$ and $S_{ree}$ from a discrete to continuous representation. Thus, the mean mixing entropy is given as follows:

(A16) $$S_{mix}\approx -kN\int_{0}^{1}\left(\left(1-x\right)ln\left(\frac{1-x}{2}\right)+xln\left(x\right)\right)f\left(x\right)dx.$$

Considering the smallness of $SD_{x}$ for large N, the integral can be approximated using Laplace’s method [36]. For the narrow distribution $f\left(x\right)$, the leading term is the value of the slowly varying function $h(x)\equiv \left(1-x\right)ln\left(\frac{1-x}{2}\right)+xln\left(x\right)$ at the mean x=ξ. A correction is obtained by expanding h(x) around x=ξ. Since $f\left(x\right)$ is symmetric around ξ, the first-order term vanishes upon integration, and only the second-order term remains. It equals the product of the second derivative ${\frac{1}{2}\left(\frac{d^{2}h(x)}{dx^{2}}\right)}_{x=\xi }=\frac{1}{2\xi \left(1-\xi \right)}$ and the variance $SD_{x}^{2}=\frac{\xi \left(1-\xi \right)}{N}$. Thus, the following holds:

(A17) $$S_{mix}\approx -kN\left(\left(1-\xi \right)ln\left(\frac{1-\xi }{2}\right)+\xi ln\left(\xi \right)\right)-\frac{k}{2},$$

According to the relation between discrete and differential entropy [37], the discrete entropy of the reaction extent is the differential entropy, plus a term involving the step width $\Delta x=\frac{2}{N}$:

(A18) $$S_{ree}\approx -k\left[\int_{0}^{1}f\left(x\right)\ln\left[f\left(x\right)\right]dx+\ln\left(\frac{2}{N}\right)\right],$$

For the Gaussian distribution, the differential entropy is equal to $\frac{k}{2}\ln\left(2\pi eSD_{x}^{2}\right)$ [37], where e is the Euler’s number and $SD_{x}$ is given by (A15). Substitution gives the following:

(A19) $$S_{ree}\approx \frac{k}{2}\ln\left(\frac{1}{2}\pi e\xi \left(1-\xi \right)N\right),$$

### Appendix A.5. Adsorption Under Nanoconfinement

In the case of N point-like molecules and N sites, the coverage x undergoes a random walk over a discrete set of values, defined by the relation $x=\frac{X}{N}$, where X=0,1,2,…,N corresponds to the number of adsorbed molecules.

The partition function of $M^{\left(x\right)}$ is given as follows:

$$Z^{\left(x\right)}=\frac{{\left(V\lambda^{-3}\right)}^{N\left(1-x\right)}}{\left(N\left(1-x\right)\right)!}\frac{N!}{\left(Nx\right)!\left(N-Nx\right)!}e^{-\frac{NxE_{ads}}{kT}}.$$

Here, $E_{ads}$ is the adsorption energy, $\frac{N!}{\left(Nx\right)!\left(N-Nx\right)!}$ gives the number of ways, disregarding order, that Nx molecules can be arranged at N adsorption sites. As above, the probability of observing a particular coverage x is as follows:

$$u\left(x\right)=\frac{Z^{\left(x\right)}}{Z},$$

Subsystem $M_{0}^{\left(x\right)}$ contains the same number of adsorbed molecules, which are separated from vacant adsorption sites. Its partition function is as follows:

$$Z_{0}^{\left(x\right)}=\frac{{\left(V\lambda^{-3}\right)}^{N\left(1-x\right)}}{\left(N\left(1-x\right)\right)!}e^{-\frac{NxE_{ads}}{kT}},$$

So that the following holds:

$$Z^{\left(x\right)}=\frac{N!}{\left(Nx\right)!\left(N-Nx\right)!}Z_{0}^{\left(x\right)}=Z_{0}^{\left(x\right)}\exp\left(kln\frac{N!}{\left(Nx\right)!\left(N-Nx\right)!}\right).$$

Similarly to Appendix A.1, the difference between the entropies of $M^{\left(x\right)}$ and $M_{0}^{\left(x\right)}$ defines the mixing entropy at reaction step x, given as follows:

$$S_{mix}^{\left(x\right)}=kln\frac{N!}{\left(Nx\right)!\left(N-Nx\right)!}.$$

The mean mixing entropies are computed using $u\left(x\right)$ and $S_{mix}^{\left(x\right)}$ according to Equation (7). For macroscopic systems, in which the probability distribution $u\left(x\right)$ sharply peaks around the mean coverage θ, the mixing entropy can be expressed by the known formula:

$$S_{mix}^{\left(TL\right)}=-kN\left(\theta ln\theta +\left(1-\theta \right)\ln\left(1-\theta \right)\right).$$

### Appendix A.6. Nanoconfined Spin 1/2 System

In the spin 1/2 system, the spin polarization x undergoes a random walk over a discrete set of values, defined by the relation $x=\frac{X}{N}$, where X=0,1,2,…,N corresponds to the number of up-spins aligned with the external magnetic field (N denotes the overall number of spins on a lattice).

The partition function of $M^{\left(x\right)}$ is given as follows:

$$Z^{\left(x\right)}=\frac{N!}{\left(Nx\right)!\left(N-Nx\right)!}e^{-\frac{Nx\Delta E}{kT}}.$$

Here, ΔE<0 is the energy difference between the spin down and up states, $\frac{N!}{\left(Nx\right)!\left(N-Nx\right)!}$ gives the number of ways, disregarding order, that Nx up-spins can be chosen from N spins. As above, the probability of observing a particular spin polarization x is as follows:

$$u\left(x\right)=\frac{Z^{\left(x\right)}}{Z}.$$

Subsystem $M_{0}^{\left(x\right)}$ contains the same number of up-spins, which are separated from down-spins. Its partition function is as follows:

$$Z_{0}^{\left(x\right)}=e^{-\frac{Nx\Delta E}{kT}},$$

So that the following holds:

$$Z^{\left(x\right)}=\frac{N!}{\left(Nx\right)!\left(N-Nx\right)!}Z_{0}^{\left(x\right)}=Z_{0}^{\left(x\right)}\exp\left(kln\frac{N!}{\left(Nx\right)!\left(N-Nx\right)!}\right).$$

Similarly to Appendix A.1, the difference between the entropies of $M^{\left(x\right)}$ and $M_{0}^{\left(x\right)}$ defines the mixing entropy at reaction step x, given as follows:

$$S_{mix}^{\left(x\right)}=kln\frac{N!}{\left(Nx\right)!\left(N-Nx\right)!}.$$

The mean mixing entropy is computed using $u\left(x\right)$ and $S_{mix}^{\left(x\right)}$ according to Equation (7). For macroscopic systems, in which the probability distribution $u\left(x\right)$ sharply peaks around the mean spin polarization P, the mixing entropy is given by the known formula:

$$S_{mix}^{\left(TL\right)}=-kN\left(PlnP+\left(1-P\right)\ln\left(1-P\right)\right).$$

The mean polarization is given as follows:

$$P=\sum_{x}u\left(x\right)x=\frac{1}{Z}\sum_{x}x\frac{N!}{\left(Nx\right)!\left(N-Nx\right)!}e^{-\frac{Nx\Delta E}{kT}}=\frac{1}{Z}\sum_{x}x\frac{N!}{\left(Nx\right)!\left(N-Nx\right)!}\lambda^{Nx},$$

where $\lambda =e^{-\frac{\Delta E}{kT}}$ and the partition function is as follows:

$$Z=\sum_{x}\frac{N!}{\left(Nx\right)!\left(N-Nx\right)!}e^{-\frac{Nx\Delta E}{kT}}=\sum_{x}\frac{N!}{\left(Nx\right)!\left(N-Nx\right)!}\lambda^{Nx}={\left(1+\lambda \right)}^{N}.$$

Consequently, the following holds:

$$P=\frac{\lambda }{N}\frac{dln\left(Z\right)}{d\lambda }=\frac{\lambda }{N}\frac{d}{d\lambda }Nln\left(1+\lambda \right)=\frac{\lambda }{1+\lambda }=\frac{1}{1+e^{\frac{\Delta E}{kT}}}.$$

Thus, P is independent of the number of spins N.
